# Moving forward the Italian nursing education into the post-pandemic era: findings from a national qualitative research study

**DOI:** 10.1186/s12909-023-04402-1

**Published:** 2023-06-19

**Authors:** Erika Bassi, Alberto Dal Molin, Anna Brugnolli, Federica Canzan, Marco Clari, Maria Grazia De Marinis, Valerio Dimonte, Paola Ferri, Federico Fonda, Loreto Lancia, Roberto Latina, Zeno Gabriele Poli, Teresa Rea, Luisa Saiani, Alvisa Palese

**Affiliations:** 1 Dipartimento di Medicina Traslazionale, Università del Piemonte Orientale, Novara, Italia; 2 Ospedale Maggiore della Carità, Novara, Italia; 3 Polo Universitario delle Professioni Sanitarie - Azienda Provinciale per i Servizi Sanitari (APSS) di Trento, Trento, Italia; 4 Dipartimento di Diagnostica e Sanità Pubblica, Università degli Studi di Verona, Verona, Italia; 5 Dipartimento di Scienze della Sanità Pubblica e Pediatriche, Università di Torino, Torino, Italia; 6 Unità di Ricerca di Scienze Infermieristiche, Università Campus Bio-Medico di Roma, Roma, Italia; 7 Dipartimento di Scienze Biomediche, Metaboliche e Neuroscienze, Università di Modena e Reggio Emilia, Modena, Italia; 8 Dipartimento di Area Medica, Università degli Studi di Udine, Udine, Italia; 9 Dipartimento di Medicina Clinica, Sanità Pubblica, Scienze della Vita e dell’Ambiente, Università degli Studi dell’Aquila, L’Aquila, Italia; 10 Dipartimento di Promozione della Salute, Materno-Infantile, di Medicina Interna e Specialistica di Eccellenza, Università degli Studi di Palermo, Palermo, Italia; 11 Azienda Ospedaliera Universitaria Integrata di Verona, Verona, Italia; 12 Dipartimento di Sanità Pubblica, Università degli Studi di Napoli Federico II, Napoli, Italia

**Keywords:** COVID-19, Lessons learned, Nursing education, Pandemic, Post-pandemic era, Qualitative study, Italy

## Abstract

**Background:**

During the CoronaVIrus-19 (COVID-19) pandemic, nursing education has been dramatically transformed and shaped according to the restrictions imposed by national rules. Restoring educational activities as delivered in the pre-pandemic era without making a critical evaluation of the transformations implemented, may sacrifice the extraordinary learning opportunity that this event has offered. The aim of this study was to identify a set of recommendations that can guide the Italian nursing education to move forward in the post-pandemic era.

**Methods:**

A qualitative descriptive design was undertaken in 2022–2023 and reported here according to the COnsolidated criteria for REporting Qualitative research guidelines. A network was established of nine Italian universities offering a bachelor’s degree in nursing for a total of 6135 students. A purposeful sample of 37 Faculty Members, 28 Clinical Nurse Educators and 65 Students/new graduates were involved. A data collection was conducted with a form including open-ended questions concerning which transformations in nursing education had been implemented during the pandemic, which of these should be maintained and valued, and what recommendations should address the transition of nursing education in the post-pandemic era.

**Results:**

Nine main recommendations embodying 18 specific recommendations have emerged, all transversally influenced by the role of the digital transformation, as a complementary and strengthening strategy for face-to-face teaching. The findings also suggest the need to rethink clinical rotations and their supervision models, to refocus the clinical learning aims, to pay attention towards the student community and its social needs, and to define a pandemic educational plan to be ready for unexpected, but possible, future events.

**Conclusions:**

A multidimensional set of recommendations emerged, shaping a strategic map of action, where the main message is the need to rethink the whole nursing education, where digitalization is embodied. Preparing and moving nursing education forward by following the emerged recommendations may promote common standards of education and create the basis on for how to deal with future pandemic/catastrophic events by making ready and prepared the educational systems.

**Supplementary Information:**

The online version contains supplementary material available at 10.1186/s12909-023-04402-1.

## Background

During the CoronaVIrus-19 (COVID-19) pandemic, the entire educational path of the health care professions underwent unprecedented modifications [1, 2] also in Italy [3].

Italy was the second country affected by the pandemic in early 2020. Universities and with them, the nursing programs, as well as the primary and secondary schools were first closed at the end of February 2020. In the first wave (February–October 2020) there were 317,409 cases and 35,918 deaths [4], which led to the imposition of the lockdown from March 9th [5] to May 3rd [6]. During the summer of 2020, the restrictions were lifted, whereas in the following autumn and winter the second and the third wave of COVID-19 occurred, resulting in 2,925,265 cases and 97,699 deaths [4]. The vaccination campaign started in late December 2020, becoming mandatory for all health-care workers, including students attending clinical rotation, and later to all citizens aged > 50 and workers. The Digital Covid Certificate’ was established as mandatory up to the 1st of January 2023 [7]. The fourth and the fifth waves occurred, respectively, in March 2021 [8] and December 2021 [9], resulting in 10,983,116 cases and 146,498 deaths on 31st January 2022 [4]. In this context, nursing programs have had to make significant adjustments from the established national and international regulations, as outlined in the European Directives. Not only the classroom teaching activities were immediately offered online in a synchronous or asynchronous manner, but also the skill labs and clinical rotations have been interrupted and transited online since the beginning of March 2020. In most nursing programs, the internship has been interrupted for long months and up to one year, and replaced by online activities, while a few have tried to maintain clinical rotations open on a voluntary basis, balancing the risk to students and the extraordinary opportunity of learning [3]. Consequently, the mandatory components of the nursing programs, including theoretical instruction, skill lab training, and clinical rotations (Table 1) have been deviated substantially.

**Table 1:** Main features of nursing programs in Italy ^a^

Main issues as defined by the law	Main description
Nursing Programs	228 several of them with more campuses
Admission and enrolment	A nationwide entry exam is mandatory for all Bachelor of Nursing Science candidates. The places available are defined by the Minister of Health and the university, with a joint law. Candidates must have at least 12 years of prior education to apply. The exam is held in September
University credits	180 credits for a total of 5,400 h
Duration	3 years
Theoretical and practical clinical training model	According to the block system, 5 months a year are used to be dedicated to theory and 6 months a year to clinical training. One month is dedicated to mandatory vacation
Lectures	Approximately 30 credits per year, 36–40 h per week. It is compulsory for students to attend at least 70% of the scheduled lectures
Skill Lab	3 credits, in simulation centres, often located at the hospital levels, for a total of 90 h. Attendance is mandatory to all expected hours of laboratory skills
Clinical training	Approximately 20 credits per year, 36 h per week. Attendance is mandatory to all expected hours of clinical rotation (1,800 h)
Number of examinations	Theoretical examinations = approximately 6 per year Practical clinical assessment = 1 per year Total = 20/21 exams Students may progress from one year to the next upon having earned the required credits and obtained positive results in the theoretical and practical examinations
Final examination	The Bachelor of Nursing Science program ends with one exam combining two sections, one awards a professional licence, the other is dedicated to the student’s thesis. The commission is composed of faculty and members of the Nursing Board

^a^According by the European Directives and the national laws [10–13]

All Italian educational systems have been exposed to a tremendous stress test demonstrating a high capacity to adapt to the pandemic circumstances; however, in the case of nursing programs, they were expected to ensure the continuity of nursing education, the expected competences, and to graduate nurses dramatically demanded by the health-care system [14, 15]. All transformations have been implemented urgently, according to the severity of the pandemic, the national, regional, and local rules, as well as the actual possibilities for hospitals to welcome students, often limited by the lack of personal protective equipment and the high risks involved [16]. Although there are limited data available, the implemented strategies have provided a valuable learning opportunity despite their unplanned implementation and poor preparation on the part of faculty members and students. Immediate positive impacts have been documented on students’ satisfaction, competences, and the length of time for students to graduate [3, 14, 17]. However, the unplanned nature of the changes, often ‘reactive’ to the public health rules gradually issued, has not facilitated the simultaneous activation of research projects to assess – in a complex situation – the effects of these changes mainly over the long term.

With the easing of restrictions, the post-pandemic phase has been started, with university systems enacting three approaches: (1) persisting in the solutions adopted during the pandemic by maintaining remote lessons [18]; (2) re-establishing the pre-existing modes of educational delivery (in the so-called ‘returning, back to normal’ approach); and (3) shaping a ‘new normality’ according to the lessons learned during the pandemic [19–21]. Nursing education has been dramatically transformed and shaped according to the restrictions that have changed over time; restoring its educational patterns as established in the pre-pandemic era while omitting to make a critical evaluation of the transformations implemented, may sacrifice the extraordinary learning opportunity that this event has offered.

Reflexivity regarding the educational changes required during the pandemic and their impact has been promoted in many countries [22–27] where several position papers on ‘what has been learned’ in the COVID-19 era have been developed, including in the Italian context, which was the first (and most severely) affected by the pandemic [28]. However, the lesson learned should be transformed into practical recommendations to address and move nursing education forward to the post-pandemic era. Establishing recommendations at the national level might also ensure harmonization [29]: the pandemic has affected each country differently, with those nursing programs located in the most affected regions having been forced to revolutionize the curriculum, thus acquiring in-depth insights into the changes needed in the future, while to a different degree/extent there are those that have been required to enact fewer changes, thus preventing an understanding of how to design the nursing education in the post-pandemic era. Moreover, a part of embodying the lesson learned, redesigning nursing education based on agreed recommendations, may promote common standards of education, and create the basis on how to deal with future pandemic/catastrophic events by making the educational systems ready and prepared [30, 31]. Therefore, the aim of this study was to identify a set of recommendations that can guide the Italian nursing education to move forward in the post-pandemic era.

## Methods

### Study design

A national-based research project ‘Lessons Learned in Nursing Education (LessonsLearNED)’ was established, aimed at identifying the recommendations for addressing the educational transition into the post-pandemic era. In this context, a qualitative descriptive study design [32] was undertaken in 2022–2023 and reported here according to the COnsolidated criteria for REporting Qualitative research [33] (Supplementary Table 1).

### Setting and participants

A network of nine Italian universities (eight public, one private) was established; five were in the North, two in the Centre and two in the South of Italy. These universities articulated from one to five campuses. At the time of the study participation, each of the nursing programs comprised from 50 to 750 students, with a total of 6135 students and a Nurse Educator:Student ratio ranging from 1:23 to 1:64. At the beginning of March 2020, the third-year students were attending their last semester of education given that the graduation was expected in November/December; the second-year students were in the middle of their education, some in classrooms and others in clinical placements; the first-year students were attending their theoretical education. All universities except one suspended the clinical rotations for an average of 170 days (the whole semester), gradually resuming them at the end of 2020 or at the beginning of 2021 (Table 2).

**Table 2:** Characteristics of the university/nursing programs involved and their campuses

University/Nursing program and Campuses	Number of Students (n)				Clinical Rotations suspension		Nurse Educators/ Students ratio (1/n)
	1 Year	2 Year	3 Year	**Total**	Period	Days (n)	
*University 1*							
Campus 1	385	220	170	775	19th Feb 2020-15th Jun 2020	117	1/38
Campus 2	155	117	96	368	Not suspended	0	1/37
*University 2*							
Campus 1	93	88	77	258	23rd Feb 2020-22nd Jun 2020	120	1/37
Campus 2	39	32	22	93	23rd Feb 2020-22nd Jun 2020	120	1/19
*University 3*							
Campus 1	73	55	49	177	Mar 2020-Oct 2020	214	NA
*University 4*							
Campus 1	205	202	200	607	Mar 2020-Oct 2020	214	1/30
*University 5*							
Campus 1	33	26	35	94	Not suspended	0	1/23
Campus 2	108	94	97	299	Not suspended	0	1/25
Campus 3	24	27	24	75	Not suspended	0	1/25
Campus 4	51	41	27	119	Not suspended	0	1/40
Campus 5	95	77	62	234	Mar 2020-15th Jun 2020	106	1/33
*University 6*							
Campus 1	416	352	562	1330	Mar 2020-Sep 2020	184	1/47
*University 7*							
Campus 1	197	131	128	456	22nd Feb 2020-15th Jun 2020	114	1/23
*University 8*							
Campus 1	108	108	108	324	Mar 2020-Nov 2020	245	1/27
Campus 2	25	25	0	50	Mar 2020-Nov 2020	245	1/25
*University 9*							
Campus 1	225	166	170	561	Mar 2020-Oct 2020	214	1/56^a^
Campus 2	71	63	59	193	Mar 2020-Oct 2020	214	1/64^a^
Campus 3	67	59	56	122	Mar 2020-Oct 2020	214	1/30^a^

Legend: *n* Number, ^a^ A Clinical Nurse Educator is present for each internship, *NA* Not appropriate, the supervision model is not based upon a Clinical Nurse Educator, *Feb* February, *Mar* March, *Jun* June, *Sep* September, *Oct* October, *Nov* November

Each university was asked to involve a purposeful sample [34] of key informants. There were eligible (a) Faculty Members, Clinical Educators, students and newly graduated, (b) who, during the pandemic, implemented (e.g. faculty members), and/or experienced (e.g., clinical educators, students/newly graduated) the changes applied in nursing education during the pandemic, and (c) willing to participate. From five to 15 participants were asked to involve at each university level, ensuring representativeness of the different key informants profile established. Potential participants were identified at the nursing program level by the researchers (see authors). In the engagement phase, they were informed of the study aims, the data collection procedures, the absolute freedom to participate on a voluntary basis and the absence of implications for any refusal. All those identified agreed to participate in the study (Table 3): 130 participants were involved, of whom 37 were Faculty Members (hereinafter, FMs, teachers, nurse educators working at the university level), 28 were clinical nurse educators (CNEs, working at the clinical level and guiding students in their rotations) and 65 were students/new graduates (STs).

**Table 3:** Characteristics of Participants

FMs	*N* = 37 (100%)
*Gender*	
Female	28 (75.7)
*Age* (years), mean (CI 95%)	45.7 (42.9–48.6)
*Education*	
Bachelor’s Degree	5 (13.5)
Postgraduate Course	4 (10.8)
Master’s Degree or more (PhD)	28 (75.7)
*Experience the Faculty role* (years), mean (CI 95%)	10.3 (8.2–12.4)
*Additional roles*	
Member of the Quality Assurance Commission	3 (8.1)
Responsible of simulation labs	2 (5.4)
Member of the Clinical Rotations Commission	2 (5.4)
Dean	1 (2.7)
Responsible of internationalization programs	1 (2.7)
**CNEs**	***N*** ** = 28 (100%)**
*Gender*	
Female	21 (75)
*Age* (years), mean (CI 95%)	42.5 (38.8–46.1)
*Education*	
Nursing Diploma	5 (17.9)
Bachelor’s Degree	5 (17.9)
Postgraduate Course	8 (28.5)
Master’s Degree or more (e.g., PhD)	10 (35.7)
*Experience as CNE* (years), mean (CI 95%)	9.43 (6.8–12.0)
**STs**	***N*** ** = 65 (100%)**
*Gender*	
Female	51 (78.5)
*Age* (years), mean (CI 95%)	23.7 (23–24.4)
*Course’s year at the beginning of the COVID-19 pandemic*	
First	43 (66.2)
Second	8 (12.3)
Third or at the point of graduation	6 (9.2)
Missing	8 (12.3)
*Previous degrees or education*	
High School diploma	47 (72.3)
Technical School diploma	10 (15.4)
Vocational School diploma	7 (10.8)
Bachelor’s Degree	1 (1.5)
*Student representative role in the University/Nursing Program board*	
Yes	24 (36.9)

Legend: *FM* Faculty member, *CNE* Clinical nurse educator, *ST* Students, *N* Population, *CI* Confidence interval

### Data collection

First, open-ended questions were developed by the research group (see authors) based (a) on their experience as educators during the pandemic period; (b) on the available literature [3, 22, 24, 35, 36], as well as (c) on the results of two preliminary focus groups, which had involved the research group (see authors) to design the research protocol submitted for the approval to the Internal Review Board. Then, a data collection form was develop including the following open-ended questions: (1) which changes in nursing education have you implemented or experienced during the pandemic period; (2) in your view/experience, which of these changes should not be maintained and which instead should be maintained and valued in the post-pandemic era according to its effectiveness, sustainability or other criteria; (3) and what recommendations should address the transition of nursing education in the post-pandemic period in light of the lessons learned. Some demographic data were also requested.

The data collection form was then sent to each participant engaged by researchers or their delegates in each partner university. In the first page of the form, they were informed of the study purposes, as well as assured of the full anonymity of the data, both with respect to the university and the participant. Then, they were asked to answer the questions in a narrative manner and send them in a sealed envelope. A deadline was established in each university and only one reminder was sent.

### Data analysis

The narratives collected were prepared verbatim in three files, distinguished for FMs, CNEs and STs (hereinafter subgroups) and by anonymizing the name of the university in all cases. The material (a total of 65 pages, font 10, line space 1) have been stored in a safe file and accessed by the analysis team (EB, ADM; AP, see authors) responsible for performing the full analysis articulated in the following four phases:

- all narratives were carefully read at each university and subgroup level to catch the main meaning.
- a content analysis was performed [37]; initially the words of the participants were extracted to in a new blank column, so that for each university we had the full list of codes regarding the recommendations to move nursing education in the post-pandemic era.
- then, the first data triangulation [38] of codes was conducted at university level: these were compared, and a final list of codes was organized in subthemes and then in themes by extracting the quotes to provide data dependability [38].
- the second data triangulation [38] was then performed at the overall level by preparing a document with all subthemes and themes emerged, comparing them according to their similarities and differences; their significant quotes were also provided. The subthemes were expressed in terms of specific recommendations by providing for each a short definition, and the exemplary quotes for each subgroup of participants, when available. The subthemes were categorized in the main themes and described as main recommendations.

Then, the list was sent to the research team, leaving them time to reflect; both written and face-to-face feedbacks were collected before, during and after meetings organized to discuss the findings and agree upon. All suggestions were considered with care. In the final stage, the list was sent again to the research team: on this occasion, findings were organized in a logical format, ordering the main recommendations emerged; feedback was requested regarding the order that was accepted by all members. In the final process, qualitative data were also integrated with a quantitative evaluation [39]: for each main recommendation, the number of participants belonging to each subgroup (e.g., FMs CNEs) who contributed with at least one quote was counted and the relative percentage calculated considering the number of total participants for each subgroup. Those themes where ≤ 25% of participants expressed a quote, were indicated with “ + ”; those cases where > 25% and ≤ 50% participants expressed at least one quote were indicated with “ +  + ”, and those cases with > 50% were indicated with “ +  +  + ”.

The data analysis process was performed by one researcher and then shared with two researchers who worked in independent fashion and then shared their findings. The research members were involved in multiple feedbacks and meetings to progressively validate the findings: they received, in advance, the data analysis performed in each stage to promote reflexivity.

### Study rigour and trustworthiness

Different strategies were implemented to ensure rigour [38]. First, to ensure credibility, the research team was prolonged engaged from the first meeting (early 2021) when the research was started; moreover, the research team was established according to its members’ authority and leadership in the field, namely their familiarity with the research context and the phenomenon, their previous research experience, as well as their skills in managing a large narrative data set.

Second, dependability [38] was ensured by involving all members in the study protocol development, with all members being consulted, and all feedback considered with care. Consistency was also ensured by documenting all changes suggested and how they were implemented. Moreover, during the data analysis, an audit trail was provided by coding and then in each step agreed upon by the research team. All modifications in the coding process were discussed to ensure a consistent interpretation throughout the analysis. Dependability was ensured by numbering (e.g. 24) each participant and the subgroup (e.g. FM1).

Third, confirmability [38] was also ensured. Specifically, reflexivity was promoted during the meetings and by collecting – in all stages of the research process – the feedback in written and/or in face-to-face meetings. Triangulation was ensured by involving several subgroups of participants, including students living in different Italian regions, thus ensuring a broad source allowing a holistic understanding of the phenomenon; moreover, research triangulation was ensured by requiring collaboration, discussion and participation by all members, which also allowed the balancing of the potential bias of individual investigators, enabling the research team to reach a satisfactory consensus level.

Fourth, the transferability [38] was promoted by checking the data saturation. That operational saturation [38], was checked by the team at each university level: most potential codes merged in the first data collection, followed by a decreasing frequency of codes when additional participants were included. However, this varied across universities, thus justifying the need to involve fewer or more participants in each subgroup. The theoretical saturation [38] was assessed in the regular meetings performed where the coding process and the variations across the data were discussed.

Moreover, the form including the four open-ended questions was piloted with five faculty members and two students, to ensure its feasibility and comprehensibility. No changes were suggested.

### Ethical issues

The study was approved by the Internal Review Board of the Udine University (Italy; approval number 144/2022). The study participation was free, and voluntary-based. No rewards were given to participants; moreover, researchers (see authors) occupying responsibility roles at the academic level (e.g. Dean) were asked to not recruit students but rather to leave them free to participate without any pressure. In this case, the data collection was performed by a member of the same university. Written consent was obtained for participation, and participants were informed that they could withdraw their consent at any time during the study. All data were processed ensuring complete anonymity [39, 40].

## Results

Participants were mostly female in all subgroups, with an average age of 45.7 years (confidence interval [CI] 95% 42.9–48) among the FMs, 42.5 (CI 95% 38.8–46.1) among the CNs and 23.7 (CI 95% 23–24.4) among students. The education level was mostly advanced, at the Master Science level or higher (28, 75.7%) among the members of the faculty, whereas the clinical educators were near half (13, 46.4%) educated at the bachelor and postgraduate course levels. Both were experts in education, given their average years of experience ranging from 10.3 (CI 95% 8.20–12.4) among the members of the faculty to 9.4 (CI 95% 6.81–12) among the clinical educators. At the time of the outbreak, students were attending mainly the first year (43, 67.2%) and around more than one-third were appointed as representatives of the community of students and were thus involved in the decision-making processes.

Nine main recommendations and 18 specific recommendations have emerged to effectively transition nursing education in the post-pandemic era. Faculty Members and Students contributed to identifying all the recommendations, while the Clinical Nurse Educators did not contribute to Recommendation Number 5 ‘Reflecting on how to effectively integrate different spaces and times of learning’ (Table 4). Moreover, while some Recommendations (Numbers 4, 7 and 9) were reported by ≤ 25% of the participants in each subgroup, the remaining were more often reported by all subgroups.

**Table 4:** Main and specific recommendations emerged to move Nursing Education in the post-pandemic era according to their source of data collection

Main and specific recommendations	Participants reporting at least one quote in each	FMs (*n* = 37)	CNEs (*n* = 28)	STs (n = 65)
**1. Acknowledging distance learning as a valuable complementary strategy**		** + + + **	** + **	** + + **
1.1 Maintaining distance learning as an opportunity to reinforce and integrate the in-classroom teaching by valuing its complementary role to the traditional learning and teaching activities 1.2 Structuring and investing in online platforms dedicated to teaching and classroom technologies for blended approaches. Investing in digital skills learning opportunities for teachers, students and administrative staff 1.3 Transiting from a free approach as an urgent solution to one established at the program level where platforms, technologies and systems are established as integral parts of the investment and learning environment 1.4 Keeping the video recording of lessons to reinforce learning and ensure usability afterwards or for students with specific needs; identifying areas in which video recordings can enrich the learning experience and outcomes and those where the video recordings are wasted				
**2. Maximizing the learning opportunities of the skill labs**		** + + **	** + **	** + **
2.1 Identifying which learning activities should be offered in the context of skill labs to anticipate the in-presence session (e.g. briefing) or as a post-lab reflection (e.g. debriefing) to maximize the time used in the labs 2.2 Strengthening the learning outcomes of skill labs by increasing their intensity offering them for small groups (no more than six students) and in limited duration, thus making these precious resources accessible, effective and impacting all students				
**3. Rethinking the clinical rotations**		** + + **	** + + **	** + **
3.1 Redesigning the clinical rotations in terms of their duration/hours and supervision models: continuing to ensure longer clinical experiences in the same setting, in 1:1 models of supervision as they guarantee greater continuity and effectiveness of learning 3.2 Redesigning where students should have their internships by considering new settings – capable of reflecting the current professional practice – going beyond the traditional units 3.3 Maintaining decentralized rotation experiences even ‘close to home’, which may retain students, and may help them to understand the needs of the community to which they belong; it may also ensure accessibility to a variety of settings and students, spreading the presence of the nursing programs across the region/area and including peripheral/remote areas				
**4. Refocusing the clinical learning aims**		** + **	** + **	** + **
4.1 Emphasizing the fundamental needs of patients, and the infection control evidence-based practices as core aims of the clinical learning processes 4.2 Reaffirming the relevance of shared clinical reasoning and multidisciplinary approaches by valuing opportunities for interprofessional clinical learning 4.3 Promoting strategies supporting students in facing complex clinical rotations, where the quality of the environments or the quality of the tutorial strategies may be suboptimal due to the disruption generated by unexpected events (e.g. a pandemic)				
**5. Reflecting on how to effectively integrate different spaces and times of learning**		** + **		** + **
5.1 Reviewing the nursing programme planning considering the different learning activities offered, by also including virtual activites, which should be visible and integrated effectively with the time devoted to lessons, skill labs and clinical rotations				
**6. Continuing with inclusive and sustainable approaches**		** + **	** + **	** + + **
6.1 Continuing in the digital transformation of nursing programs to facilitate inclusiveness and sustainability, which may affect proximity, timeliness and personalized approaches to students 6.2 Increasing a paperless approach in each point of education, by reflecting on what can be transferred digitally by revising nursing program regulations				
**7. Creating and sustaining the modern student community**		** + **	** + **	** + **
7.1 Researching and experiencing new practices capable of creating the 'academic community' of students in which their physical and virtual presence are facilitated and enriched				
**8. Being ready: have a pandemic education plan**		** + + **	** + **	** + + **
8.1 Developing an educational pandemic plan at the national and/or local levels, to harmonize the decision, and provide actions capable of maintaining the continuity of nursing education and its quality.				
**9. Redefining and launching new research priorities**		** + **	** + **	** + **
9.1 Identifying the research priorities focused on nursing education transition in the post-pandemic era, on which to promote networks by involving all nursing programs and investing resources				

Each main recommendation has been supported by quotes. Therefore, for each subgroup of participants, the quotes were counted, and the relative percentage calculated considering the total number of participants. In those cases where ≤ 25% of participants expressed a quote, these were indicated with ‘ + ’; in those cases where > 25% and ≤ 50% participants expressed at least one quote, there were indicated with ‘ +  + ’, and in those cases > 50%, we indicated these with ‘ +  +  + ’

Legend: *FMs* Faculty members, *CNEs* Clinical nurse educators, *STs* Students

### Recommendation 1: acknowledging distance learning as a valuable complementary strategy

According to the Italian law and the European Directives [10], Italian nursing programs have always required a ‘compulsory attendance’ (Table 1). Only a few universities had started the technological transition path before the pandemic in limited activities such as seminars or individual meetings. The nursing programs have experienced a forced digital transition, triggering the acquisition of new technologies and skills. It was also a precious opportunity to understand the value of face-to-face learning activities and the equally important subsidiary role of online ones. Initiating a reflection to recognize and integrate the contribution of distance learning as a complementary activity, reinforcing or supporting the complex learning processes, could ameliorate the student’s experience and outcomes, alongside the value of the face-to-face learning activities, which should be focused on core aspects of nursing education.

Therefore, the following specific recommendations have emerged to move nursing education forward in the post-pandemic era:

1.1 Maintaining distance learning as an opportunity to reinforce and integrate the in-classroom teaching by valuing its complementary role to the traditional learning and teaching activities.

*FM11 - I consider the possibility of implementing traditional training with a blended learning method as positive, which allows for flexible, multi-method education thus capable of satisfying multiple learning needs and styles.*

*CNE11 - Implementing integrated teaching by enhancing which scientific knowledge requires the mandatory presence in the classroom and which can be provided remotely.*

*ST1 - Online lessons (…), I personally prefer face-to-face lessons even if I believe that for many students it is useful for any extra-university commitments to be able to follow lessons from home. So, I think it would be useful to keep this method, but with the main course of the lessons requiring to be attended...*

1.2 Structuring and investing in online platforms dedicated to teaching and classroom technologies for blended approaches. Investing in digital skills learning opportunities for teachers, students, and administrative staff. The faculty members should also be prepared to design effective learning opportunities remotely, and this requires investments in their teaching abilities.

*FM1 – distance learning, through the use of the XXXX platform (...), represented the opportunity to acquire new skills and new tools that I consider useful to continue to use, in situations agreed with the students, and to integrate other strategies*.

*CNE3 - I consider it useful that the training process in the digital field for teachers, which began during the COVID period, is being continued*.

*ST12 - I think that all this set of new knowledge and technological tools must be maintained over time, and I also think it is appropriate to use them for activities that cannot be carried out in person*.

1.3 Transiting from a free approach as an urgent solution to one established at the program level where platforms, technologies and systems are established as an integral part of the investment and learning environment.

*FM12 - use of an online platform, e.g. XXXX, to collect material delivered/indicated to students during the course year, I suggest keeping it to facilitate the students learning.*

*CNE21 - Once the emergency is over, the university could therefore identify the resource to use* .

*ST12 - The XXXX platform has been used a lot during the COVID-19 pandemic, I think it's a very comfortable and versatile tool…*

1.4 Keeping the video recording of lessons to reinforce learning and ensure usability afterwards or to help students with specific needs; identifying areas in which video recordings can enrich the learning experience and outcomes and where they are wasted.

*FM36 - I believe that there are some positive aspects that can be maintained: the possibility of recording lessons (in addition to face-to-face lessons) with free use by students*.

*CNE27 - One could think of recording the lectures so that they can be listened to at any time, especially by those who are absent. One could think of the possibility of interventions by other teachers/health professionals working in other realities, including abroad*.

*ST13 - The recorded lessons mode is very useful for focusing on the large amount of information that is said within a lesson and for reproducing passages*.

### Recommendation 2: maximizing the learning opportunities of the skill labs

Skill labs have been well established to achieve cognitive, technical, or relational/affective skills; these have been integrated into the journey of students, usually between the classroom and the clinical rotations, with in-group guided reflection activities before and after each session, all of them conducted ‘in-presence’ (Table 1). Several programs have also been equipped with advanced simulation centers offering learning activities to groups of students (e.g. 8–10 or more). During the pandemic, even these activities have been profoundly revised: usually offered in hospital settings that have become inaccessible, they have been transformed into online activities; when possible, they were carried out in person for very few students (e.g. a maximum of six) and for a limited duration in accordance with the restrictions. To support each other, universities located in the country have also shared resources online by pooling clinical cases, supervisors and platforms. There was learnt that not all the learning processes enacted in the skill labs require a ‘physical’ presence, given that some activities can also be offered remotely. By reducing the number of students and the duration of the lab sessions, there were offered also high-intensity sessions, making time, space and resources more efficient to maximize the learning outcomes. Thus, there is a need to identify the possible contribution of distance strategies in the context of skill labs, valuing virtual learning; there is also a need to identify in which circumstances and for which aims the involvement of small groups of students and for a short time in 'intensive labs' can maximize the expected learning outcomes. Therefore, the following specific recommendations have emerged to move nursing education forward in the post-pandemic era:

2.1 Identifying which learning activities should be offered in the context of skill labs to anticipate the in-presence session (e.g. briefing), or as a post-lab reflection (e.g. debriefing) to maximize the time used in the labs.

*FM28 - Personally, I would not abandon any of the strategies adopted during the pandemic period to integrate simulation laboratory activities (…) because they offer different possibilities for students to collaborate, communicate and exchange opinions, make learning more interactive and captivating...*

*CNE22 - once the pandemic is over, I would suggest continuing with the implementation of this type of strategy with the ultimate goal of increasing students’ preparedness before the labs*.

*ST17 - As regards the cognitive lab [those aimed at discussion clinicals scenarios], the online manner favoured comparison and participation. …these were also used to ‘anticipate’ the session... making the learning multidimensional …*

2.2 Strengthening the learning outcomes of skill labs by increasing their intensity, offering them for small groups (no more than six students) and for limited duration, thus making these precious resources accessible, effective and impacting all students.

*FM6 - The number of students per single session decreased (…). It gave the tutor the opportunity to have the right time and the right attention (…). It made it possible to easily activate discussion (…). I would maintain this tutor/student ratio in the skill labs in the future.*

*ST5 - Dividing students into small groups was more effective*.

### Recommendation 3: rethinking the clinical learning rotations

Before the pandemic, each nursing program established its own pattern regarding the eligible units, the expected number of rotations; the number of first-, second- and third-year students eligible for each unit and the tutorial model (e.g. involving all the nurses of the facility or clinical nurse educators, in a 1 to 1 or 2 ratio). These patterns were similar across the nursing programs (Table 1).

The limitations imposed by the pandemic have led to a major revision of these traditional patterns: new units have been considered as a place for rotations; to limit units being overcrowded, the students use of public transport/travelling, a strong decentralization strategy has been enacted, offering rotations in health services/units close to the students’ home. Moreover, longer time spent in each unit was offered (9 weeks or more) to avoid unnecessary rotations and the risk of contamination between settings. In addition, to avoid unnecessary exposure, limiting student contacts with the team, and to facilitate tracing, a 1:1 supervising model was introduced. Furthermore, the quality of the environments as good places to learn was not considered as a criterion for deciding the eligibility for rotations, given the diffuse disruption of the nursing practice in all units; in contrast, due to the need to ensure the clinical rotations to all students in a safe environment, the safety criteria were set as the first criterion. These aspects appear to have revolutionized the pre-pandemic rotation patterns requiring all a reflection. Therefore, the following specific recommendations have emerged to move nursing education forward in the post-pandemic era:

3.1 Redesigning the clinical rotations in terms of their duration/hours and supervision models: continuing to ensure longer clinical experiences in the same setting, in a 1:1 model of supervision as they guarantee greater continuity and effectiveness of learning.

*FM14 - During the pandemic period, each ward had a reduced number of students and each student was followed up by a supervising nurse in one-to-one mode. I find this breakdown of students to be more formative… This choice, sustainable from a pedagogical point of view, has also met with the appreciation of the students who have experimented with it*.

*CNE24 - The main change regarding the traineeship of the students was certainly the support of only one nurse. In this way we were able to ensure driving continuity.*

*ST9 - The longer internship experiences allowed me to better adapt to the place where I was, allowing me to achieve a certain degree of autonomy and security*.

3.2 Redesigning where students should have their internships by considering new settings – capable of reflecting the current professional practice – going beyond the traditional units.

*FM16 - in the pandemic we have placed students in several contexts, including innovative ones with reduced numbers for each context. They turned out to be welcoming internship environments with interesting and effective learning opportunities.*

*CNE6 - I think the experience in this area should be maintained for part of the internship (…) given that depriving them of this experience would mean losing a learning opportunity.*

*ST6 - The possibility of carrying out an internship in a new setting (...) allowed me to understand more deeply the nursing care provided to people with a specific health problem.*

3.3 Maintaining decentralized rotation experiences even ‘close to home’ that may retain students, may help them to understand the needs of the community to which they belong; it may also ensure accessibility to a variety of settings and students, spreading the presence of the nursing program across the region/area and including peripheral/remote areas.

*FM12 - We experienced more internship opportunities in community and outpatient services since the first year of the bachelor’s degree. I suggest maintaining this.*

*CNE11 - We have introduced internship programs in community settings where they weren't previously available or, in some circumstances, didn't exist at all.*

*ST9 - As regards the internship experiences, I really appreciated the possibility of staying close to home, when possible, since each location has its own peculiarities and offers different training opportunities.*

### Recommendation 4: Refocusing the clinical learning aims

During the COVID-19 outbreak, students learning has been focused on some essential aims that were sometimes overlooked in the pre-pandemic phase, as for example: the full and constant implementation of safety standard precautions (e.g. use of PPE); the possibility of taking care of each patient as a whole in all needs, in the so-called clustering of interventions, *'I enter the room and take care of all the patient*'*s needs’* to avoid cross-contamination. Multidisciplinary work was also strongly introduced: in many departments, there were nurses and doctors from different specialties with frequent discussion meetings or the search for consultancy to address new problems. Students also learned the value of the internship as a ‘precious’ experience, to be ‘desired’, not taken for granted, where facing several difficulties requires coping and stress managing abilities. Efforts should be made to keep the value of the internship high, continuing to offer multidisciplinary experiences based on good standards of practice. Also, efforts to support students’ resilience and coping strategies in managing complex situations (e.g. units dramatically changing in their routines) are required.

Therefore, the following specific recommendations have emerged to move nursing education forward in the post-pandemic era:

4.1 Emphasizing the fundamental needs of patients, the infection control evidence-based practices, as core aims of the clinical learning processes.

*FM33 - All of this has made us wonder a lot, as health professionals, about the quality of life of people in their last days and has confronted us with difficult ethical choices.*

*CNE3 - focus on the dignity of the person: during the pandemic era, in the COVID wards there was a lack of contact, not being able to convey empathy with gestures or with a smile, the limitation of access by relatives... The action of the nurses used a common standard, such as dressing, undressing, hygiene and hand washing, and the students had a single reference, and not, as happens among our realities, in which the standardization of assistance is a limitation.*

*ST1 - Raise students' awareness of the need for discussion with the patient and/or relative as they are subjected to forced isolation.*

4.2 Reaffirming the relevance of shared clinical reasoning and multidisciplinary approaches by valuing opportunities for interprofessional clinical learning.

*FM16 - In the pandemic phase we have increased occasions of shared/multidisciplinary reasoning never seen before…*

*CNE17 - keeping in mind the need to also reflect on gaps triggered by the changes imposed by the pandemic.*

*ST61 - Nursing education helped me a lot in the pandemic period to understand and reflect how to behave in the community.*

4.3 Promoting strategies supporting students in facing complex clinical rotations, where the quality of the environments or the quality of the tutorial strategies may be suboptimal due to the disruption generated by unexpected events (e.g. a pandemic).

*FM36 - …Avoiding ‘hiccup suspension’ of student internships to prevent negative impact on skills.*

*CNE12 - Very often the internships are subjected to delays … but this certainly did not help the student's training.*

*ST45 - Many of us have had to change several wards due to the increase in the number of hospitalized COVID patients (…). Changing different working environments (…) it seemed to me that I was not able to learn in an optimal way.*

### Recommendation 5: Reflecting on how to effectively integrate different spaces and times of learning

The traditional didactic approach called the ‘block system’, characterized by a linear alternation of the main learning activities, has been considered an effective method in nursing education (Table 1). Thus, students have always been engaged in classroom activities to learn theoretical aspects, then in skill lab sessions, and in the units for their clinical experience. This model basically separated the different learning moments by creating a logical sequence of three spaces and times. With the introduction of distance learning, it has also become necessary to imagine a ‘fourth time and space’, dedicated to virtual activities. The ‘fourth space and time’ should be carefully planned and integrated and not managed individually by each teacher/student, to ensure consistency, prevent issues related to prolonged video terminal exposure, fatigue, stress and/or ineffectiveness due to overlapping competitive learning activities. An effective integration of all different activities may also prevent excessive loads and requires a remodelling of the overall planning of the educational path.

Therefore, the following specific recommendation has emerged to move nursing education forward in the post-pandemic era:

5.1 Reviewing the nursing program planning, considering the different learning activities offered by also including that virtual learning activities, which should be visible and integrated effectively with the time devoted to lessons, skill labs and clinical rotations.

*FM18 - I would increase the methodologies integrating distance learning itself to reduce stress for students and competitive learning activities (offered by different teachers at the same time)*

*CNE – No quotes*

*ST16 – With the advent of the pandemic, changing the times from morning to afternoon allowed for better organization of time/study.*

### Recommendation 6: continuing with inclusive and sustainable approaches

During the pandemic, universities wondered how to ensure the inclusion of students in economic difficulty, coming from remote areas with poor connectivity, with special learning needs not easily assessable and manageable online. They have also dealt with sustainability: for example, in their ability to accommodate students in adequate and ventilated classrooms; to ensure that everyone had access to face-to-face lessons when possible; to adapt the lessons and clinical rotations avoiding crowded public transport or limiting excessive student flows in the university corridors or in the units. To access internships, priority was given to third-year students, neglecting first-years; sustainable solutions have also been identified to ensure exam sessions by offering them online, ensuring the continuity of education during the lockdown, which in turn also led to reduced consumption of some resources, such as papers. Some of these issues have been addressed by the university as a whole; others, only from the nursing programs, in which sustainability has been further threatened by the lack of nurse educators left on the campuses, often called back into clinical units. Different strategies have ensured inclusiveness and sustainability, two aspects on which it is necessary to keep a high level of attention.

Therefore, the following specific recommendations have emerged to move nursing education forward in the post-pandemic era:

6.1 Continuing with the digital transformation of nursing programs to facilitate inclusiveness and sustainability, which may affect proximity, timeliness, and personalized approaches to students.

*FM4 – What it brought to light, however, were some situations of socio-economic difficulties such as, for example, that of taking distance learning exams so as not to impact on the costs of reaching the university location. All this to ensure greater academic inclusiveness.*

*CNE18 – However, online teaching allowed all students in home isolation to be able to access teaching at any time and therefore to be able to continue their studies.*

*ST48 – I think the pandemic has taught us many things and to appreciate and use appropriately all the materials we have available, such as the Internet, PCs (…) and saving resources thus increasing sustainability … I believe it is appropriate that all teaching and assessment activities are maintained and carried out in this way [remotely, ed.] ensuring a green future.*

6.2 Increasing a paperless approach at each point of education, by reflecting on what can be transferred digitally by revising nursing programs’ regulations.

*FM10 - the XXX platform can also be used for in-person, where the students use their own devices, and the faculty members can directly supervise… this would also be beneficial for students participating in Erasmus programs overseas.*

*CNE10 - written exams on the XXX platform with immediate return of results reduces the possibility of grading errors.*

*ST4 - for example, we are used to sign our presence in the classroom every day on white paper with all the names of the students printed on. I suggest finding/using an app and coding the student’s presence – certainly a much simpler and more functional tool than the signature sheet. I would suggest keeping it.*

### Recommendation 7: Creating and sustaining the modern student community

According to the compulsory attendance, the classrooms of the nursing programs, as well as the skill labs and the rotations have always been frequented by many students (Table 1), becoming a place for interaction and exchange. The lockdown and social distancing restrictions emptied universities for a long time; during these hard times there was rediscovered the importance and value of sociability, that of relationships and the students’ community. However, there is a widespread perception of a disintegration of the student community; moreover, there is a diffuse perception that students have some difficulties in creating and living in their academic community and this may affect their emotional well-being, their reciprocal support during education, and their future. Students today are together physically but also 'virtually': therefore, there is a need to facilitate and support them with new strategies. Finally, since nursing is a relationship-based profession, it will be necessary to understand how the restrictions experienced by students have influenced the basic relational skills on which professional ones are grafted.

Therefore, the following specific recommendation has emerged to move nursing education forward in the post-pandemic era:

7.1 Researching and experiencing new practices capable of creating the 'academic community' of students in which their physical and virtual presence are facilitated and enriched.

*FM16 - working groups were suspended during the pandemic or were run exclusively online. I feel they have been missed so much and need to be taken up 100%.*

*CNE17 – there were lack of the social aspect of the relationship, from the lack of empathy and human contact that only a face-to-face course can provide.*

*ST3 - we have hardly experienced the university environment.*

### Recommendation 8: Being ready, have a pandemic education plan

Italy has faced several dramatic events (e.g. earthquakes) in the past but never of a magnitude similar to the pandemic in terms of diffusion and persistence over time. The pandemic has found the nursing programs substantially unprepared: some reacted immediately, while others have activated less immediate responses. However, while with the online teaching, the continuity of theoretical education was guaranteed, the clinical rotations were all interrupted, and their re-initiation was complex for the required interactions with hospitals. Some nursing programs have also faced tensions with their students for whom it has not been easy to understand the rotation suspension of different durations across campuses according to local pandemic trends, the resources available and the restraints imposed by the local health facilities.

The education of future nursing generations must be considered an essential service of the country. Therefore, each nursing program should prepare and have an emergency plan to be agreed inside of the university and with the health-care facilities where students are expected to attend their clinical placements to preserve the continuity and quality of clinical rotations in difficult times.

Therefore, the following specific recommendation has emerged to move nursing education forward in the post-pandemic era:

8.1 Developing an educational pandemic plan at the national and/or local levels, to harmonize the decision, and providing actions capable of maintaining the continuity of nursing education and its quality. Specifically, actions that should be included in the pandemic plan can be the following:
  Assessing the resources and constraints of students in order to also define a pandemic educational plan in light of the difficulties and constraints (e.g. access to technology) and opportunities (e.g. technological skills).
  Adapting the predefined pandemic education plan to the emerging situation by involving students and faculty members to consider resources/opportunities and perceived constraints, in a co-constructive process.
  Approving as soon as possible the adapted pandemic educational plan with the academic community (faculty members/students) to ensure high adherence, coherence of each teacher's choices and full understanding of the decision undertaken.
  Preferring synchronous methods of distance learning to ensure interaction and continuous contact with students. Asynchronous video recordings should be used as a complementary learning strategy.
  Promoting alliances/strategies to keep the clinical rotations as an irreplaceable learning strategy. Leaving students the freedom to attend the clinical rotations, providing them with appropriate training regarding the risks and the use of appropriate protective equipment; supporting students in the learning process that may trigger stress.
  Avoiding intermittent rotations (e.g. suspensions) unless these are justified (e.g. suspected contagion); providing immediate alternatives by providing students with the reason for the suspension.
  Ensuring continuity of the assessment system (written online, interviews, reports) considering the benefits and risks related to the use of the technology (online assessments) and the additional stress caused by uncertainty (for example malfunction); identifying for each learning outcome the most effective manner to evaluate its achievement.
  Promoting an effective supportive system for students and faculty members capable of mitigating the stress, the difficulties and the challenges encountered in maintaining a high quality of teaching and learning processes; the well-being of the entire academic community should be cared for and supported in its influences on student’s learning outcomes.
  Identifying and implementing an evaluation system capable of detecting issues and to inform the decision regarding potential critical consequences on the expected learning outcomes.

*FM24 - Some campuses have stopped internships until May 2021!*

*CNE4 - We must avoid interrupting internships. Continuous outbreaks in the non-COVID wards have seen students stop and restart their internship several times.*

CNE12 - For the future, considering the ever-increasing knowledge and availability of PPE and training courses, I would re-evaluate the possibility of an internship, even a short one, for 3rd-year students, including internships in the COVID areas.

*ST62 - The asynchronous online lessons (…) did not allow for a complete exchange between students and professors, for this reason I would suggest abandoning them…. I found it difficult to follow the lessons recorded by the teachers, since an ongoing exchange/clarification was not possible.*

*ST41 - Doing an internship half in one department and half in another department was not very constructive; therefore, I would not keep it as an option because the quality of the internship is lost.*

*ST45 - Many of us have had to change several wards due to the increase in the number of hospitalized COVID patients (…). Changing different working environments (…) it seemed to me that I was not able to learn in an optimal way.*

### Recommendation 9: re-defining and launching new research priorities

Research should accompany the expected transitions in nursing education to understand their effectiveness, short- and long-term impact, and its added value in the expected competences of students. Therefore, doctorate programs, academicians and educators should consider this emerging need in their daily research agenda.

Therefore, the following specific recommendation has emerged to move nursing education forward in the post-pandemic era:

9.1 Identifying the research priorities focused on nursing education transition in the post-pandemic era, on which to promote networks by involving all nursing programs and investing resources.

*FM16 - during the pandemic, the hours of clinical rotations were reduced … (are they all really needed?).*

*CNE23 - the mentoring activity has been roughly maintained even via online … without any data regarding its effectiveness.*

*ST7 - Investigating the limitation of the non-verbal interaction between students and teachers due to the mask: now that the teacher is allowed not to use the mask, the relationship with the class is greatly improved.*

## Discussion

Our research project was aimed at critically analyzing the transformations implemented during the pandemic across Italy to identify the recommendations based to address the nursing educational transition into the post-pandemic era. Similar reflections have been promoted in other countries [22–28] mainly highlighting ‘what has been learned’; we have tried to transform the lesson learnt into practical recommendations to address the nursing education development.

Our research team engaged a large number of Faculty Members, Clinical Educators and Students/graduates to reflect on the revolutionary changes taking place in different regions of our country, where different strategies have been implemented according to the severity of the pandemic. Italy was affected dramatically by the pandemic and nursing education was strongly affected, altering its well-established profile defined by the national laws and European directives [11–13]. Forcing the rules and implementing new strategies to keep vital nursing education have provided a unique occasion for learning and building up some recommendations to inform the transition in the post-pandemic era, the future national policies and also those internationally established.

The nine main recommendations emerged with their several specific lines of actions – suggesting three main reflections. First, the post-pandemic model of nursing education in Italy should be developed on a ‘new normality’ according to the lessons learned during the pandemic [19–22], preventing any return to the pre-pandemic norm or by keeping the changes implemented in extraordinary circumstances in the daily practice of the nursing programs. The intermediate phase between the end of the pandemic and the new normality requires a strong alliance across universities to shape the new future.

Second, the several recommendations that have emerged affect the entire nursing curriculum, not just a part of it (for example, classroom activities): overall these recommendations are transversally influenced by the role of digitization, in its various forms, as a complementary and strengthening strategy for face-to-face teaching as an ‘added value’. The inclusion of digital transformation will require a revision of the study program not only to identify in which areas and to what extent students and faculties might benefit from its contribution, but also to develop the digital skills expected among students and teachers. The pandemic experience has certainly contributed to strengthening and sometimes building a set of digital skills among teachers and tutors that will need to be maintained and further developed over time to accompany the digital transition [41], even with respect to simulation, traditionally offered in face-to-face laboratories, but which has also become virtual [42–44]. Also, in the case of team-based learning, nursing students report satisfaction and impact on clinical reasoning skills with the transition to virtual rather than face-to-face meetings [45]. In this context, an assessment of the funds needed to support the digital transition of study programs is necessary.

Third, in weighting the recommendations by counting the number of participants in each subgroup, some were suggested by several participants (e.g. ‘Acknowledging distance learning as a valuable complementary strategy’; ‘Rethinking the clinical rotations’), whereas others were suggested by a few, students excluded (e.g. ‘Reflecting on how to effectively integrate different spaces and times of learning’). The value of the distance learning has been recognized as complementary not as a substitution for the presence of the students in the academic environment, the value of which has been confirmed. In contrast, the clinical rotations should be redesigned: findings highlighted the need to re-discuss and calibrate the duration, settings, aims and the supervision models of the clinical placements, which represent a central element of the nursing education. The role of decentralized internship units, often not included in the rotations network, needs to be discussed, according to the potential impact to include students coming from rural areas. In addition, the role of outpatient clinics, which have not been included as clinical rotations for years because there were preferred high-intensive experiences mainly in hospitals, should also be discussed. Assessing the potential value of the outpatient settings (e.g. in the oncological field, chronicity) including in rural areas to avoid a priori exclusions or centralization of traineeships in large hospitals is recommended to make education sustainable, considering the increased need of nursing students and that of other professions. In this light, the concept of inclusiveness and sustainability have emerged aa central in the post-pandemic area, in line with already established worldwide agendas [46].

All participants, but to a lesser extent, recommended attention should be paid towards the student community; the pandemic has left them alone and disrupted, impoverished in relational skills in 'presence' and very rich in those at a distance (e.g. social). The strategies to be implemented to help them to ‘become a community’ both in the university settings but also in the perspective of the future profession, represent another priority [47, 48].

There are also recommendations suggested by a few participants, and not by students. This is the case with regards to the need to identify strategies to better integrate the space and times of virtual learning into teaching programming, until now organized around three main activities, all requiring the students’ physical presence – namely, in the classroom, in the skill labs and in the clinical placements. Students may have the ability to learn in different simultaneous environments (physical, in classroom, and digitally, online); however, this multitasking approach may affect the cognitive loads threatening the learning outcomes [49].

Participants suggested the need to define a pandemic educational plan to be ready for unexpected but possible events, as also suggested by international experiences [50]. Nursing education should not be at risk of being interrupted: its continuity should be maintained as an essential service of the country. Therefore, being prepared is also an ethical imperative [36, 51].

### Strengths and limitations of the study

To our best knowledge, no previous studies have been conducted in this field in the Italian context. Emerged findings and insights in the form of recommendations based on a comprehensive understanding of the issues, can support the transition of nursing education in the post-pandemic phase. In fact, by collecting rich, detailed data through open-ended questions and non-standardized measures, qualitative research can provide a nuanced understanding of a particular issue, which can then inform decisions regarding complex phenomenon.

However, the study has some limitations. Data were collected at the end of the pandemic period and not during the early stages; this could have introduced recall bias with respect to some teaching/didactical strategies introduced and later dismissed. Furthermore, we have included universities located in different regions of the country that have experienced different intensity of the pandemic waves: some pre-existing structural differences across universities may have influenced their pandemic response but also the lessons learned. Different subgroups of participants were engaged, but not the administrative staff of universities who may have offered another perspective on the phenomenon; in addition, it would have been interesting to include nurses in executive roles who recruited our new graduates to understand their preparedness in pandemic times. The data analysis considered a very complex approach, first at the level of each university and then at the whole level: although there were numerous rigour strategies introduced, the background and the experience of the researchers could have influenced the interpretation of the data.

## Conclusions

As the pandemic era draws to a close and a new phase—commonly referred to as the ‘post-pandemic era’—begins, this research was aimed at contributing to identify the main priorities to guide Italian nursing education in this next phase. To achieve this goal, different perspectives have been considered, from faculty members to students and new graduates, valuing their experiences and perceptions, as lived in different universities located across Italy.

The post-pandemic model of nursing education in Italy should be based on a ‘new normality’ according to the lessons learned during the pandemic, preventing the return to the pre-pandemic routine on the one side and the consolidation of the changes implemented in extraordinary circumstances as those lived by the country. A multidimensional set of recommendations emerged, shaping a strategic map of action, where the main message is the need to rethink the whole of nursing education, whereby digitalization is embodied. Preparing and moving nursing education forward by following the emerged recommendations may promote common standards of education and create the basis on how to deal with future pandemic/catastrophic events by making the educational systems ready and prepared. 

## Supplementary Information

**Additional file 1: Supplementary Table 1.** COnsolidated criteria for REporting Qualitative research Checklist [33].

## Data Availability

Since they were conducted in Italian rather than English, the full-text interviews used in the study are not publicly accessible, but they are available from the corresponding author upon reasonable request.
