# Supplementary material for: Moving forward the Italian nursing education into the post-pandemic era: findings from a national qualitative research study
Source: BMC Med Educ. 2023 Jun 19;23:452. doi: 10.1186/s12909-023-04402-1 (PMC10280977; doi:10.1186/s12909-023-04402-1)
Supplement: Supplementary file 1 — Additional file 1: Supplementary Table 1. COnsolidated criteria for REporting Qualitative research Checklist [33]. [file 12909_2023_4402_MOESM1_ESM.docx]

**Supplementary Information**

**Supplementary Table 1.** COnsolidated criteria for REporting Qualitative research Checklist [33]

| **Topic** | **Item No.** | **Guide Questions/Description** | **Reported on Page No.** |
| --- | --- | --- | --- |
| **Domain 1: Research team and reflexivity** | | |  |
| *Personal characteristics* | | |  |
| Interviewer/facilitator | 1 | Which author/s conducted the interview or focus group? | There were developed a data collection form that was administered by a member of the research team (see authors) at the university level |
| Credentials | 2 | What were the researcher’s credentials? E.g. PhD, MD | Mostly of them were educated at the advanced levels, with long experience in research |
| Occupation | 3 | What was their occupation at the time of the study? | All were appointed at the University level as a Faculty Members |
| Gender | 4 | Was the researcher male or female? | In composing the research team, there were considered the need to balance the gender to ensure inclusiveness |
| Experience and training | 5 | What experience or training did the researcher have? | Mostly of them have previous experience in both qualitative and quantitative research (see their publications in indexed journals) |
| *Relationship with participants* | | |  |
| Relationship established | 6 | Was a relationship established prior to study commencement? | There were a professional or a pedagogical relationship established before and during the study; in the case of students in order to ensure freedom, the data collected involved a delegate (not the Dean, when member of the research team) |
| Participant knowledge of the interviewer | 7 | What did the participants know about the researcher? e.g. personal goals, reasons for doing the research | None in particular |
| Interviewer characteristics | 8 | What characteristics were reported about the inter viewer/facilitator? e.g. Bias, assumptions, reasons and interests in the research topic | There was reported only the main intent of the study and the collaborative nature of the study design at the national level. No other issues were reported |
| **Domain 2: Study design** | | |  |
| *Theoretical framework* | | |  |
| Methodological orientation and Theory | 9 | What methodological orientation was stated to underpin the study? e.g. grounded theory, discourse analysis, ethnography, phenomenology, content analysis | A qualitative descriptive study design |
| *Participant selection* | | |  |
| Sampling | 10 | How were participants selected? e.g. purposive, convenience, consecutive, snowball | A purposeful sample was adopted |
| Method of approach | 11 | How were participants approached? e.g. face-to-face, telephone, mail, email | Each university was left free to decide the best strategy to approach the potential participants (via phone, via email) according to the internal rules and routines |
| Sample size | 12 | How many participants were in the study? | A total of 130 participants |
| Non-participation | 13 | How many people refused to participate or dropped out? Reasons? | None |
| *Setting* | | |  |
| Setting of data collection | 14 | Where was the data collected? e.g. home, clinic, workplace | The data was collected with a form sent to the participants allowing them to reflect and find out the best moment and place to fill in the open-ended questions |
| Presence of non-participants | 15 | Was anyone else present besides the participants and researchers? | NA |
| Description of sample | 16 | What are the important characteristics of the sample? e.g. demographic data, date | The main characteristics of the setting and that of participants are reported in Table 1, 2 and 3 |
| *Data collection* | | |  |
| Interview guide | 17 | Were questions, prompts, guides provided by the authors? Was it pilot tested? | The data collection form was piloted on a preliminarily fashion and no changes were requested.  The semi-structured questions addressed the reflection |
| Repeat interviews | 18 | Were repeat inter views carried out? If yes, how many? | No |
| Audio/visual recording | 19 | Did the research use audio or visual recording to collect the data? | No |
| Field notes | 20 | Were field notes made during and/or after the inter view or focus group? | No |
| Duration | 21 | What was the duration of the inter views or focus group? | Around 20 minutes as emerged in the pilot phase |
| Data saturation | 22 | Was data saturation discussed? | The saturation was discussed among the research team as reported in the data analysis and in the rigor and trustworthiness section |
| Transcripts returned | 23 | Were transcripts returned to participants for comment and/or correction? | No |
| **Domain 3: analysis and findings** | | |  |
| *Data analysis* | | |  |
| Number of data coders | 24 | How many data coders coded the data? | Three as reported in the data analysis |
| Description of the coding tree | 25 | Did authors provide a description of the coding tree? | Yeas, see in the data analysis. The coding tree is available from authors upon request |
| Derivation of themes | 26 | Were themes identified in advance or derived from the data? | The themes and sub-themes were derived from the data |
| Software | 27 | What software, if applicable, was used to manage the data? | None |
| Participant checking | 28 | Did participants provide feedback on the findings? | No |
| *Reporting* | | |  |
| Quotations presented | 29 | Were participant quotations presented to illustrate the themes/findings? Was each quotation identified? e.g. participant number | Yes, see the findings section |
| Data and findings consistent | 30 | Was there consistency between the data presented and the findings? | Yes, see the findings section and the Table 4 |
| Clarity of major themes | 31 | Were major themes clearly presented in the findings? | See the themes, in the findings section and in the Table 4 |
| Clarity of minor themes | 32 | Is there a description of diverse cases or discussion of minor themes? | See the subthemes, in the findings section and in the Table 4 |

NA, Not Applicable
